# Role of p53/miR-155-5p/sirt1 loop in renal tubular injury of diabetic kidney disease

**DOI:** 10.1186/s12967-018-1486-7

**Published:** 2018-05-30

**Authors:** Yue Wang, Zong-ji Zheng, Yi-jie Jia, Yan-lin Yang, Yao-ming Xue

**Affiliations:** Department of Endocrinology and Metabolism, Nanfang Hospital, Southern Medical University, Guangzhou, Guangdong China

**Keywords:** Diabetic kidney disease, HK-2, miRNA, miR-155-5p, Sirt1, p53, Autophagy

## Abstract

**Background:**

Diabetic kidney disease is a renal microvascular disease caused by diabetes, known as one of the most serious and lethal complications of diabetes. Early renal hypertrophy is the main pathological feature, which gradually leads to the deposition of glomerular extracellular matrix and tubulointerstitial fibrosis, eventually developing irreversible structural damage to the kidneys. Autophagy is a cell self-homeostatic mechanism that is activated under stress conditions and may serve as a protective response to the survival of renal fibrogenic cells. MicroRNA (miRNA) network may be involved in the regulation of fibrosis. The purpose of this study is to assess how miRNAs regulate diabetic kidney disease and autophagy and fibrosis in renal proximal tubular cells under high glucose conditions.

**Methods:**

Human renal proximal tubular (HK-2) cells were exposed to high glucose in vitro. Bioinformatic analysis was used to select the candidate gene for potential target regulation of miR-155, Sirt1. ATG5, ATG7 is the key to autophagosome formation, regulated by Sirt1. p53 regulates miR-155 expression as a transcription factor. MiR-155 overexpression and inhibition were achieved by transfection of miR-155 mimic and inhibit to evaluate its effect on Sirt1 and autophagy and fibrosis markers. Dual luciferase reporter assays were used to confirm the direct interaction of Sirt1 with miR-155. Overexpression and inhibition of Sirt1 gene were achieved by transfection of Sirt1 plasmid and Sirt1 si to observe its effect on P53. Chip assay experiments confirmed the direct regulation of P53 on miR-155.

**Results:**

Under high glucose conditions, miR-155 was detected in HK-2 cells in concentration gradient, increased expression of p53 and down-regulated expression of sirt1 and autophagy-associated proteins LC3II, ATG5 and ATG7. Dual luciferase reporter assays indicate that miR-155 can target its binding to the Sirt1 3′UTR region to reduce its expression. Under high glucose conditions, over expression of miR-155 decreased the expression of LC3-II and ATG5 in HK-2 cells, while inhibition of miR-155 reversed this effect. Using chip assay testing in HK-2 cells, we demonstrated that p53 binds directly to miR-155.

**Conclusions:**

The signaling axis of p53, miR-155-5p, and sirt1 in autophagic process might be a critical adapting mechanism for diabetic kidney injury.

## Background

Diabetic kidney disease (DKD) is one of the most common and devastating complications of diabetes [1]. It is also the high risk factors of end-stage renal disease (ESRD) and significantly increases the risk of diabetes Patient’s mortality rate. Diabetes has become a global health problem, for the prevention of complications, postpone the development of diabetic kidney disease is particularly important. Despite the control of blood glucose, blood pressure and blood lipids, such as proteinuria and other comprehensive treatment, but the current clinical efficacy is still poor. Because of the complicated metabolic disorder in patients, it is more difficult for DKD to progress to end-stage treatment, which is one of the important reason of death in patients [2]. Therefore, looking for an earlier and more effective new target for prevention and treatment of diabetic nephropathy, preventing or delaying the progress of DKD is a problem that the medical profession has so far failed to solve, exploring the pathogenesis of DKD in search of new and effective prevention and treatment methods has important medical and social research value [3, 4].

MicroRNA is a single-stranded, non-coding RNA about 22-24 nucleotides in length that can degrade or inhibit protein translation by specifically binding to the 3 ‘non-coding region of the target gene mRNA. The binding mechanism is very flexible and complex [5]. Recent research, miR-155-5p is observed significantly increased in Diabetic kidney disease patients’ kidney tubules [6] and can aggravate renal fibrosis in patients with acute kidney injury [7]. The mechanism of microRNA expression changes is focused on the control of transcriptional activity by the binding of transcription factors to promoters. It is noteworthy that there is literature describing that p53 promotes the expression of miR-155-5p in tumor cells and tissues [8]. Therefore, we hypothesize that the p53/miR-155-5p loop may be involved in the regulation of the injury process of renal tubular cells under high glucose conditions.

MicroRNA regulates gene expression by inhibiting the translation process, cutting and degrading mRNA, and reducing mRNA stability. We tried to find the target gene for miR-155-5p [9]. There is growing evidence that Sirt1 is under the control of microRNAs such as miR-199 [10], miR-200 [11] and miR-22 [12]. We predicted the existence of binding sites between the miR-155-5p and Sirt1 3′UTR regions by the online bioinformatics analysis (TargetScan) [13].

Sirt1 is the most famous member of the silent information regulator 2 family,when we detect patients with diabetic nephropathy (DKD) or study DKD animal models, we find that the expression of Sirt1 in renal cells tends to decrease, and further studies have found that increasing the expression of SIRT1 can be constructed well. Kidney protection is provided in animal models with DKD disease [14, 15]. SIRT1 can exert anti-apoptosis, anti-oxidation and anti-inflammatory effects in cell injury and protect cells by regulating mitochondrial biogenesis, autophagy and metabolism in response to cellular energy and redox state. Specifically, SIRT1 can directly de-acetylate key proteins (Atg) of autophagy, such as Atg5, Atg7, and Atg8, to remove them from the inhibitory state, thereby promoting autophagy [16]. At the same time, SIRT1 can also deactivate transcriptional activity of p53, thereby inhibiting its function.

In summary, we investigated the mechanism of p53/miR-155-5p/Sirt1 loop in high glucose-induced tubular epithelial cell injury in vitro.

## Methods

### Cell culture

10% FBS (Gibco, NZL) was supplemented in MEM (Gibco, Invitrogen) and human renal proximal tubule (HK-2) cells were cultured at 37 °C and 5% The medium was changed every 2 days, waiting for HK-2 cells to grow to 60% and then starved for 24 h in a medium supplemented with MEM (Gibco, Invitrogen) supplemented with 2% FBS (Gibco, NZL). Group 1: (normal glucose) normal glucose concentration (5.5 mmol/l) medium as a control. Group 2: normal cell culture medium supplemented with high glucose (11 mM) medium. Group 3: normal cell culture medium supplemented with high glucose (20 mM) medium. Group 4: normal cell culture medium supplemented with high glucose (30 mM) medium as high glucose stimulation group. All cells were incubated with medium for 72 h. To observe the expression of miR-155-5p in all groups. And to observe the expression of Sirt1, P53, autophagy and fibrosis related factors in both 1 and 4 groups.

### Quantitative real-time RT-PCR

We extracted total RNA from HK-2 cells using a phenol–chloroform extraction protocol and used to determine the purity and concentration of RNA after NANODROP 2000 DEPC water was zeroed. After RNA was uniformly diluted to 100 ng/μl, cDNA was reverse transcribed. Quantitative real-time RT-PCR was performed on a LightCycler 480 (Roche, CH). PCR reaction system for 1 μl cDNA, 0.2 nM of each primer, 3.6 μl SBRE then RNase-free water to make a total amount of 10 μl. Using GAPDH as an internal control, the resulting CT values for Sirt1, p53 and autophagy-related markers of fibrosis. The primers we used are showed in Table 1.

**Table 1:** Sequences of primers for qRT-PCr in this study

Gene	Sequences	
Sirt1	Sense	5′-AGTTCCAGCCGTCTCTGTGT-3′
	Antisense	5′-CTCCACGAACAGCTTCACAA-3′
Atg5	Sense	5′-TGTGCTTCGAGATGTGTGGTT-3′
	Antisense	5′-ACCAACGTCAAATAGCTGACTC-3′
Atg7	Sense	5′-ACCCAGAAGAAGCTGAACGA-3′
	Antisense	5′-CTCATTTGCTGCTTGTTCCA-3′
Col-1	Sense	5′-TCAAGACACGTTCCCGTGAG-3′
	Antisense	5′-GCCAACTTCTCCAGCGGTA-3′
GAPDH	Sense	5′-GAACGGGAAGCTCACTGG-3′
	Antisense	5′-GCCTGCTTCACCACCTTCT-3′
miR-155-5p	Sense	5′-GCCTCCAACTGACTCCTACA-3′
	Antisense	Universal reverse primer (Tiangen, Beijing, China)
U6	Sense	5′-CTCGCTTCGGCAGCACA-3′
	Antisense	Universal reverse primer (Tiangen, Beijing, China)

### Western blotting

Using western blotting to detect the expression level of Sirt1 and P53 and the expression of autophagy-related proteins LC3II and p62. Briefly, the extracted protein samples were mixed with SDS-PAGE and boiled in boiling water for 5 min to denature. The isolated protein was then electrophoretically transferred to a polyvinylidene difluoride (PVDF) membrane (0.45 mm) (Millipore, USA). Incubate with 5% skim milk in TBS for 1 h to block non-specific proteins. The primary antibodies used include the following: Sirt1, p53, LC3 II, p62, FN, Col-1 and GAPDH. After incubating for 2 h at room temperature with pre-prepared rabbit secondary antibody (1: 5000; Santa Cruz biotechnology, USA) and mouse secondary antibody (1: 5000; Santa Cruz biotechnology, USA) and using chemiluminescence (ECL) Imaging Analysis System Exposure photography. Relative protein stripe density through the image inspection software quantity one for testing and analysis.

### Transfection

1. To effect changes in miR-155-5p expression, HK-2 cells were treated with miR-155-5p mimic/inhibit (bought from ribobio Biotechnology company, Guangzhou China) using both LipofectamineTM 3000 as a transfection reagent and a non-sense strand negative control (NC) as a control. Briefly, cells were starved overnight in 12-well plates at 60% density prior to transfection. Lipofectamine 3000 was mixed with 50 μl Opti-MEM, meanwhile, miR-155-5p mimic/inhibit was mixed with 100 μl Opti-MEM. The two mixtures were then mixed for 5 min and then added to the cell culture medium and left to incubate for 48 h at 37 °C in 5% CO_2_. Collect cells for protein or total RNA extraction. q-PCR was used to validate the up-regulation of miR-155-5p. After verification, the expression of Sirt1, p53, ATG5, LC3II, p62 and fibrosis related genes were detected by western blot and q-PCR.
2. To achieve changes in Sirt1 expression, HK-2 cells were treated with Sirt1 plasmid or Sirt1 si using LipofectamineTM 3000 as transfection reagent and non-sense strand negative control (NC) as controls. Similar to the experiment described in the previous phase, cells were starved overnight in 6-well plates at 60% density prior to transfection. Lipofectamine 3000 was mixed with 125 μl Opti-MEM while Sirtl plasmid or Sirtl si was mixed with 250 μl Opti-MEM at a 1 μg target dose (plasmid group supplemented with 10 μl P3000TM Reagent/well). The two mixtures were then mixed for 5 min and then added to the cell culture medium for further 48 h at 37 °C in a 5% CO_2_ incubator. Protein or total RNA was extracted from the cell collection plate for subsequent experiments. The changes of Sirt1 expression were verified by western blot and q-PCR. The expression of p53, ATG5, LC3 II, p62 and fibrosis related genes were observed after validation.

### Luciferase reporter gene assay

We used PCR to amplify the miR-155-5p and Sirt1′ 3′ UTR binding sites (primer: forward: 5′-CCGCTCGAGTGTAATAATTGTGCAGGTACAG-3′; reverse: 5′-ATTTGCGGCCGCAAAGTTAGTGTTGAGTTTGTAC-3′) from HK-2 cell genomic DNA. The amplified Sirt1 3′UTR fragment was inserted into the psi-Check2 vector (Ribobio, Guangzhou, China) respectively, using the *Xho*I and *Not*I restriction sites. HK-2 cells were setted in 24-well plates at 60% density treated by MEM medium containing 2% Gibico fetal calf serum for 24 h. After this, cells were co-transfected with psi-Check2 (0.5 μg) constructed with Sirt1 and miR-155-5p binding sites with miR-155-5p mimics/inhibitors, MEM medium containing 30 nm d-glucose and 10% Gibico fetal bovine serum was used to groom. Cells were harvested 48 h after transfection, dual luciferase assay system was used to analyze the luminescence.

### Chip assay

The presence of p53 binding sites in the promoter region of miR-155-5p was predicted by software. Using a Chip assay, in brief, cells were treated with 1% formalin and sonicated to collect soluble chromatin supernatants at 14,000*g* for 10 min at 4 °C. Anti-p53 antibodies (Santa Cruz Biotechnology, USA.) Overnight, mouse IgG immunoprecipitated as a negative control. Immune complexes were washed and DNA samples were obtained with a QIAquick Gel Extraction Kit (QIAGEN, Ger). We use qRT-PCR to analysis the recovered DNA by using primers containing the miR-155-5p promoter region and the P53 binding site. (primer: forward: 5′-CCGCATGTGCATACACAAAC-3′; reverse: 5′-CATTTAGGATACTACTGATAAATC-3′).

### Statistical analysis

All of the experimental data obtained in this study were entered using Excel spreadsheet software. Use SPSS 20 software to accomplish statistical analysis. Statistical results were expressed as mean ± standard error of mean. Use independent means t test to compare the mean of the two samples. Use One-way ANOVA to compare multiple groups. P < 0.05 was statistically significant.

## Results

### High glucose stimulates the general measurement of HK2 cells

**Fig. 1:**
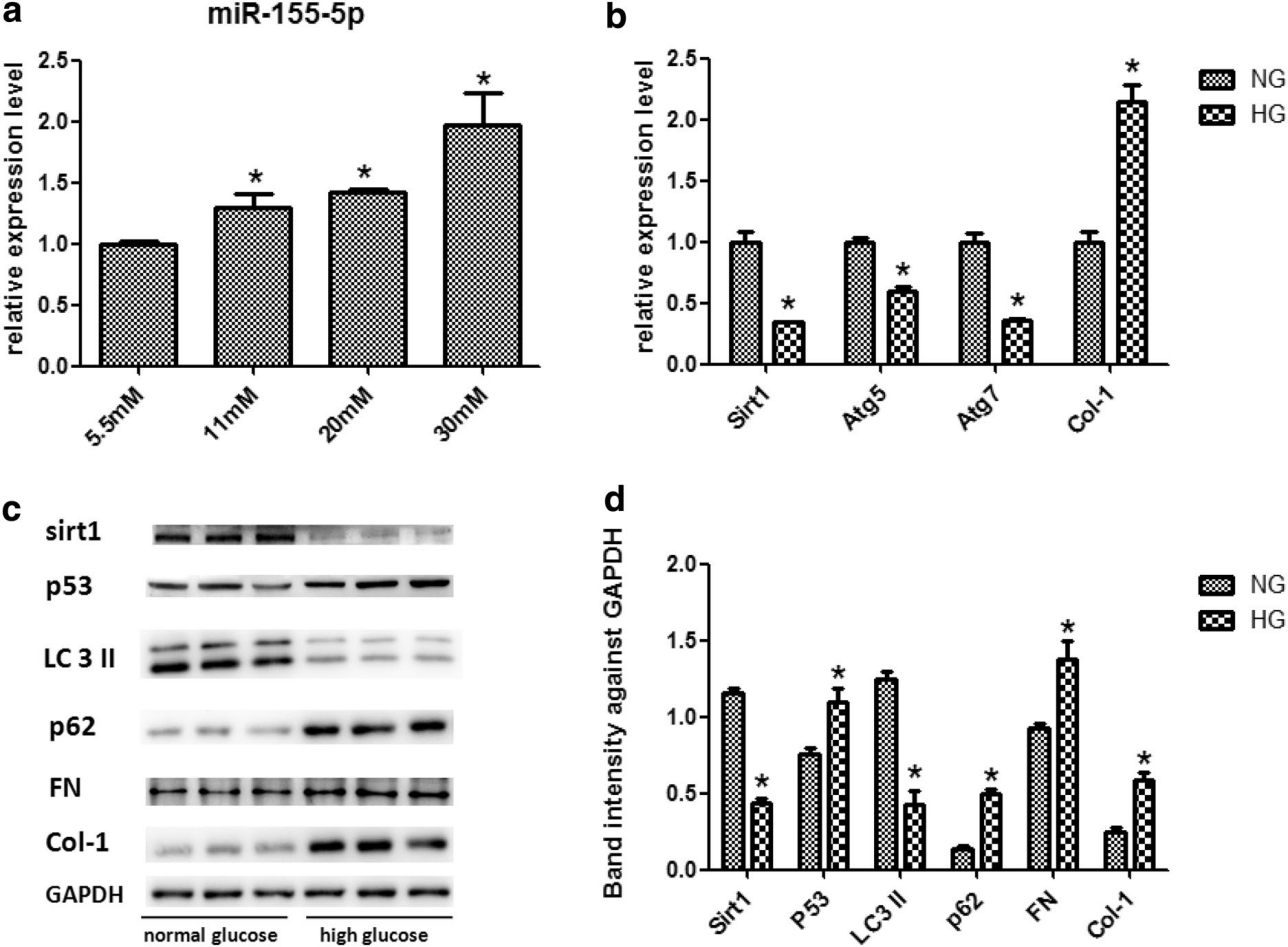
High glucose can promote the expression of miR-155-5p in HK-2 cells in concentration gradient, **a** inhibit the expression of Sirt1, autophagy-related index, promote P53 and promote the expression of fibrosis molecules. **b** QRT-PCR and **c** western blot with **d** quantitative analysis of Sirt1, P53, LC3 II, p62, FN and Col-1 expression in HK-2 cells treated with high glucose (30 mM) for 72 h. Results are presented as mean ± SEM of three independent experiments. *P < 0.05 vs NG, *NG* normal glucose (5.5 mM)

The expression of miR-155-5p is significantly increased in patients with diabetic nephropathy. Explore what role miR-155-5p plays in renal tubular injury. We chose HK-2 cells as an in vitro experimental model. The test was divided into four groups, the normal glucose (5.5 mM) group and the gradient high glucose group (11 mM, 20 nM, 30 mM). After culturing the HK-2 cells for 72 h, total RNA and protein were extracted for analysis. We found that the expression level of MiR-155-5p in HK-2 cells was gradually up-regulated with the increase of glucose concentration (11 mM, 20 nM, 30 mM) compared to the cells treated with normal glucose (5.5 mM). Sirt1 expression was significantly inhibited by high glucose (30 mM). P53 expression was increased by stimulating high concentrations of glucose (30 mM). Western blot results showed that after high glucose stimulation (30 mM), we observed changes in autophagy-related expression, with a significant decrease in LC3 II and a significant increase in p62. After high glucose stimulation (30 mM), the fibrosis index FN, Col-1 also increased significantly (Fig. 1).

### MiR-155-5p directly regulates Sirt1

**Fig. 2:**
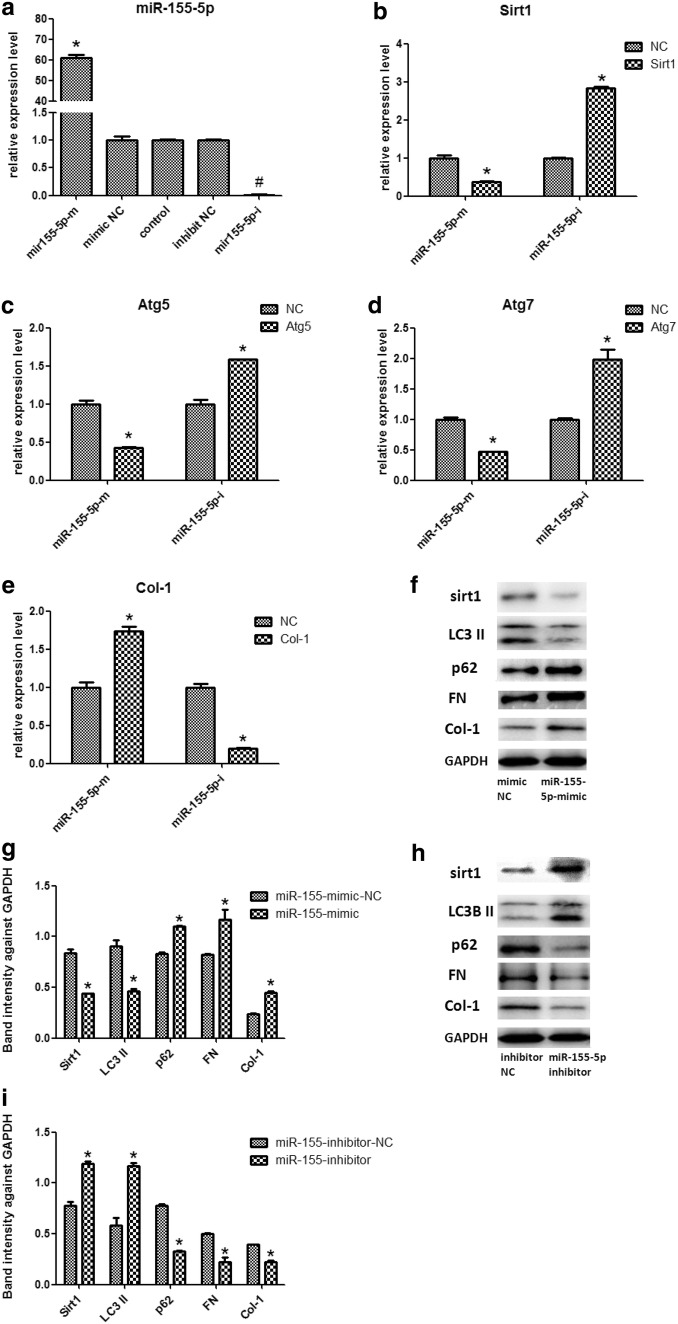
MiR-155-5p promote Sirt1-inhibited fibrosis in HK-2 cells via inhibiting autophagy activity. **a** miR-155-5p mRNA expression was detected by qRT-PCR analysis in HK-2 cells transfected with miR-155-5p mimics/inhibit. **b**–**e** miR-155-5p inhibit decreased the expression levels of Sirt1, Atg5, Atg7, Col-1, on the contrary, miR-155-5p inhibit increased the expression levels of Sirt1, Atg5, Atg7, Col-1 by qRT-PCR and **f** the influence of miR-155-5p mimics/inhibitor on Sirt1, LC3II and p62, FN, Col-1 expression level measured by western blot with (**f**–**i**) quantitative analysis. Results are presented as mean ± SEM of three independent experiments. *P < 0.05. *miR-NC* miRNA negative control, *miR-155-5p-m* miR-155-5p mimics, *miR-155-5p-i* miR-155-5p inhibitor

**Fig. 3:**
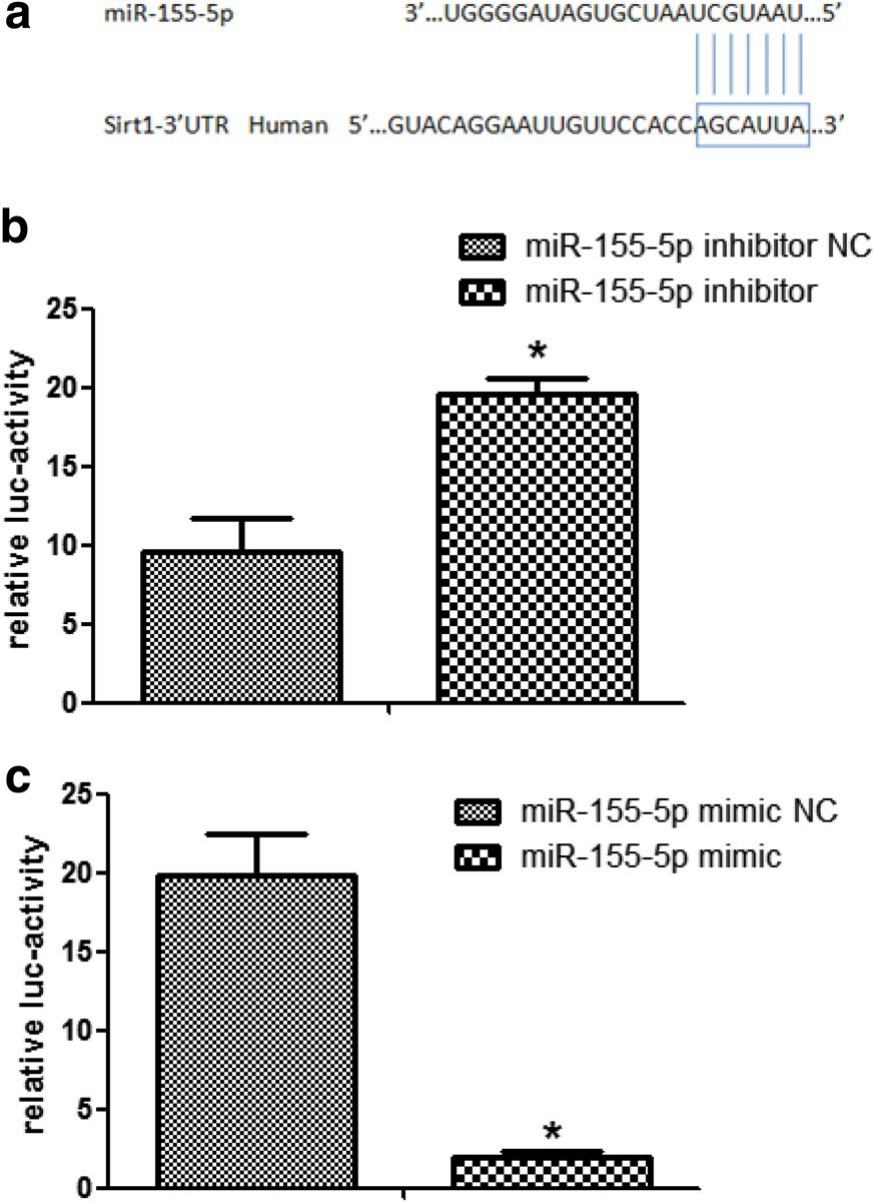
The Sirt1 3′UTR is regulated by miR-155-5p. **a** MiR-155-5p and its putative binding sequence in the 3′UTR of Sirt1. Luciferase assay of HK-2 cells co-transfected with miR-155 inhibitors (**b**) or mimics (**c**) and the luciferase reporter. The experiments were performed in triplicate. The data are expressed as the mean ± SEM. *P < 0.05. *miR*-*155*-*i* miR-155 inhibitor, *miR*-*inhibit*-*NC* miRNA inhibit negative control, *miR*-*155*-*m* miR-155 mimics, *miR*-*mimic*-*NC* miRNA mimic negative control

Aberrant expression of miR-155-5p was confirmed in HK-2 cells, and we then verified whether Sirt1 was directly regulated by miR-155-5p expression. We change miR-155-5p expression level by Transferring miR-155-5p mimic or inhibit into HK-2 cells. After transferring miR-155-5p mimic in HK-2 cells, miR-155-5p levels were significantly increased by 61.006-fold, whereas the Sirt1 mRNA and protein expression was diminished contrast with the NC group (Fig. 2a). The reverse trend was discovered in HK-2 cells after miR-155-5p inhibit. As shown (Fig. 2), levels of miR-155-5p are reduced by a factor of 100, while expression of Sirtl mRNA and protein is increased. Subsequently, we constructed psi-Check2, a dual luciferase plasmid carrying the Sirt1 fragment and co-transfected with miR-155-5p mimic and inhibit. The results showed that the psi-Check2 fluorescence ratio co-transfected with miR-155-5p mimic significantly decreased, whereas the reverse significantly increased, suggesting that Sirt1 and miR-155-5p binding (Fig. 3).

### Sirt1 direct regulation P53 in high glucose cultured HK-2 cells

**Fig. 4:**
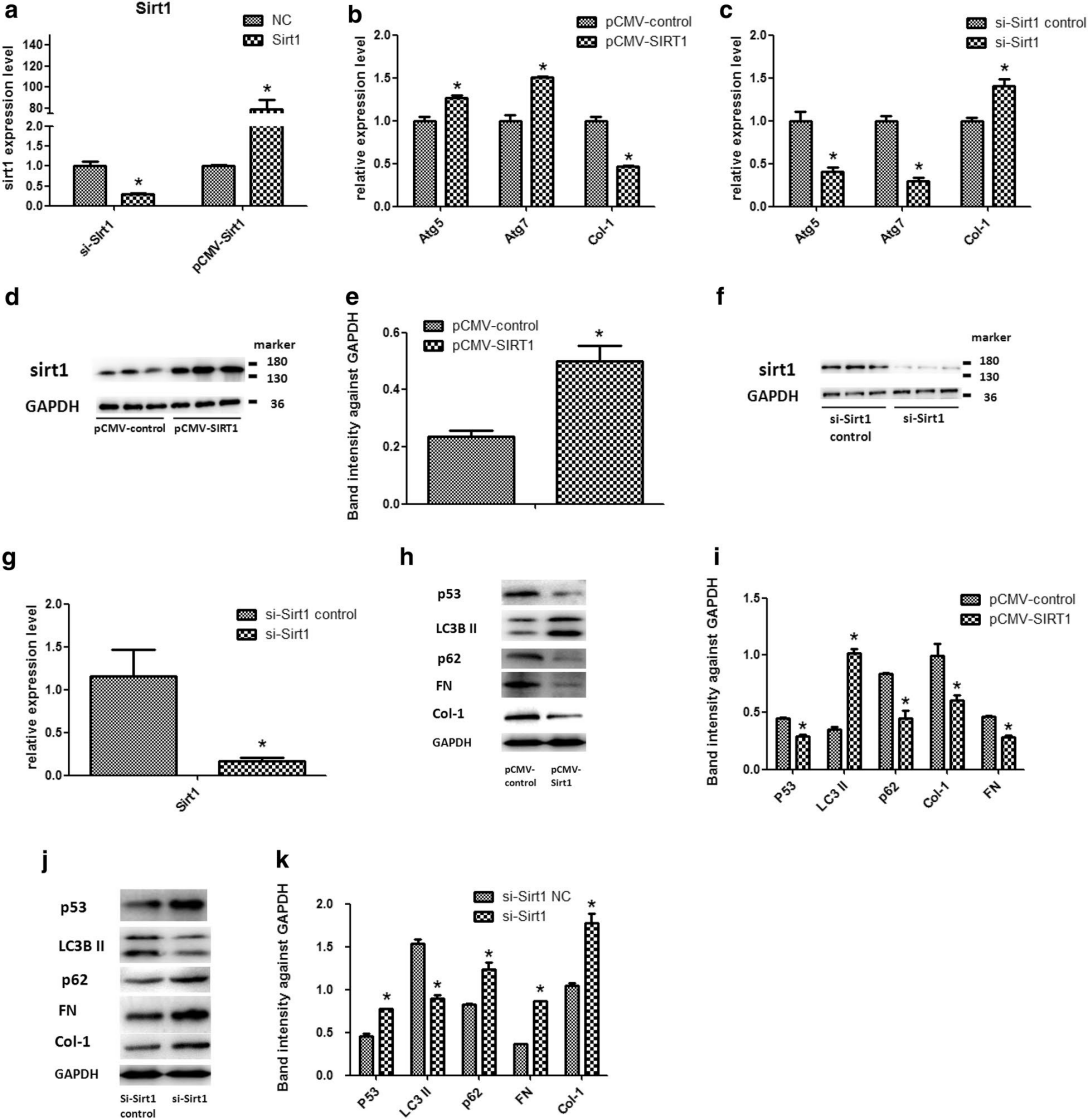
The Sirt1 regulate expression of P53. **a** Sirt1 mRNA expression was detected by qRT-PCR analysis in HK-2 cells transfected with pCMV-Sirt1 plasmid, and confirmed by **d** western blot with **e** quantitative analysis. **b** Sirt1 over expression increased the levels of Atg5, Atg7, LC3 II and decreased the expression levels of P53, P62, FN, Col-1 by qRT-PCR and **h** western blot with **i** quantitative analysis. **a** Sirt1 mRNA expression was detected by qRT-PCR analysis in HK-2 cells transfected with si-Sirt1, and confirmed by **f** western blot, **g** quantitative analysis. **c** Inhibition of expression of Sirt1 gene increased the levels of P53, P62, FN, Col-1 and decreased the expression levels of Atg5, Atg7, LC3 II by qRT-PCR and **j** western blot with **k** quantitative analysis. The experiments were performed in triplicate. The data are expressed as the mean ± SED. *P < 0.005. *pCMV-Sirt1* Sirt1 over expression, *pCMV-control* Sirt1 over expression negative control

To confirm that Sirt1 directly inhibits P53 expression in high glucose cultured HK-2 cells, we transfected HK-2 cells with LipofectamineTM 3000 to overexpress or suppress the Sirt1 gene to determine the effect of Sirt1 on P53. As shown in Fig. 4, Sirt1 plasmid transfection confirmed that Sirt1 gene was overexpressed and its expression was increased by 79.12-fold. After Sirt1 gene overexpression, Sirt1 gene expression was significantly inhibited by P53 expression. By q-PCR and Western blotting, we observed that P53 expression was significantly reduced after Sirt1 overexpression in high glucose conditions, and this effect was reversed after Sirt1 was inhibited. The data demonstrate that in high glucose treated HK-2 cells sirtl can directly inhibit P53 expression (Fig. 4).

### P53 binds to miR-155-5p

**Fig. 5:**
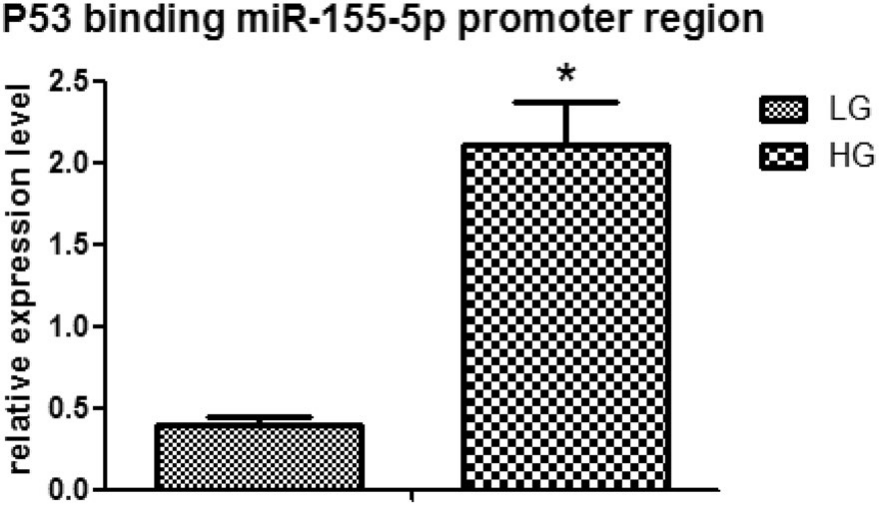
High glucose promote P53 binds to miR-155-5p promoter region. P53 and miR-155-5p promoter binding region was detected by qRT-PCR analysis DNA from Chip assay in HK-2 cells treated by High glucose (30 mM) group complete with normal glucose (5.5 mM) group. The data are expressed as the mean ± SEM. *P < 0.005. *LG* normal glucose (5.5 mM), *HG* high glucose (30 mM)

We use chip assay to assess whether p53 directly regular miR-155-5p gene expression in HK-2 cell cultured by high glucose. High glucose can stimulate P53 and miR-155-5p promoter region binding. The phenomenon was then confirmed by qRT-PCR analysis, as shown in Fig. 5, with a significant up-regulation of P53 and miR-155-5p promoter binding sites after high glucose stimulation, with statistical significance. It shows that P53 pathway-upregulated miR-155-5p expression exists in high glucose group. We demonstrated that P53 can direct increase the expression of miR-155-5p (Fig. 5).

## Discussion

The early manifestations of diabetic nephropathy are renal hypertrophy, glomerular and tubular basement membrane thickening and other obvious pathological features. As the disease progresses, it gradually evolves into glomerular extracellular matrix accumulation of tubulointerstitial fibrosis and eventually Irreversible damage to the kidney structure. Some studies have suggested that the changes in tubules may precede the glomeruli, suggesting that tubules may be a key player in the development of DKD [17]. Therefore, renal tubular injury has become an important area of DKD research in recent years, and autophagy dysfunction, especially impaired autophagy, plays an important role in renal tubular injury [18]. Previous studies have found that Sirt1 is a protective factor of tubular cells and that Sirt1 expression was increased in both human and animal models of diabetic nephropathy (DKD) and confirmed to be protective in DKD kidneys [19]. Specifically, it can exert its deacetylation, freeing the key proteins of autophagy from acetylation inhibition, thereby promoting autophagy activity and improving adaptability to stress environments. Our experiments also confirmed that high glucose stimulation can induce renal tubules. Cell Sirt1 expression was decreased, autophagy was impaired, tubular cell injury was induced [20], and P53 was also involved [21] as downstream regulator of sirt1 [22, 23]. In contrast, in a mouse model of diabetes, improving tubular autophagy can reduce tubular cell oxidative stress and reverse renal tubular injury in diabetic mice [24]. Due to the complex mechanism of renal tubular injury in DKD, the study of renal tubular injury, especially tubular autophagy, has become the core of DKD tubular injury.

According to the analysis software predictive search, we found that the target binding site of miR-155-5p on Sirt1 3′UTR region is very interesting. miR-155-5p is a transcription product of tumor gene BIC. It has been widely studied in the tumor field in the past. This year it was found to have a promoting effect on lung and liver fibrosis, and was gradually introduced into the study of renal fibrosis. In 2015, it was discovered that MiR-155-5p promotes fibrosis of proximal tubule cells and EMT by modulating TGF-β1 under hypoxic conditions [7]. In 2017, Baker et al. [6] found that the expression of miR-155-5p was significantly increased in the renal tubules of patients with diabetic nephropathy, which contributed to its important role in the development of diabetic nephropathy. In the same year, another investigator discovered that the expression of miR-155-5p was significantly elevated in the serum of patients with chronic kidney disease and nocturnal hypertension, suggesting that miR-155-5p played a role in renal tubular disease and even renal tubular injury in diabetic nephropathy, important role. In our study, we demonstrated for the first time that the expression of miR-155-5p increased with the increase of high glucose concentration in HK-2 cells. miR-155-5p is transcriptionally regulated by p53 and participates in the regulation of cell cycle, cell growth, differentiation and apoptosis. We speculate that miR-155-5p up-regulation may inhibit Sirt1, activate P53 and form a positive feedback loop. Although it has been reported that miR-155-5p is involved in the promotion of renal fibrosis under hypoxic conditions, the existence of the p53/miR-155-5p/Sirt1 loop and its mechanism of action in renal tubular injury and renal fibrosis in diabetic nephropathy clear. In this study, we demonstrated for the first time that the expression of miR-155-5p increased with the increase of high glucose concentration in HK-2 cells. miR-155-5p is transcriptionally regulated by p53 and participates in the regulation of cell cycle, cell growth, differentiation and apoptosis. We speculate that miR-155-5p up-regulation may inhibit Sirt1, activate P53 and form a positive feedback loop. Although miR-155-5p is reported to be involved in the promotion of renal fibrosis under hypoxic conditions, the presence of the p53/miR-155-5p/Sirt1 loop and its role in renal tubular injury and renal fibrosis are clear in diabetic nephropathy. In this study, we investigated the presence and function of p53/miR-155-5p/Sirt1 loop in renal tubular cells stimulated by high glucose, providing a new idea for the mechanism of diabetic tubular injury.

## Conclusion

- miR-155-5p is a microRNA that was found to have significantly increased renal tubular specificity in patients with diabetic nephropathy last year.
- Our data broaden the scope of the study of miR-155-5p, confirming its role in high glucose-induced tubular injury.
- Our data revealed, for the first time, the existence of a new signaling loop p53/miR-155-5p/Sirt1 that miR-155-5p is involved in regulating,this is a new mechanism of high glucose-induced renal tubular damage and provides evidence that miR-155-5p over expression inhibits the sirt1-regulated autophagy pathway and serves as a therapeutic target for diabetic nephropathy.

## Abbreviations

DKD: diabetic kidney disease
HK-2 cells: human renal proximal tubular cells
Sirt1: sirtuin type 1
p53: tumor protein 53
LC3: autophagy marker light chain 3
p62: autophagy substrate protein 62
ATG5: autophagy related genes 5
ATG7: autophagy related genes 7
FN: fibronectin
Col-1: collagen I
GAPDH: glyceraldehyde-3-phosphate dehydrogenase
NC: negative control
EMT: epithelial–mesenchymal transition
3′UTR: 3′untranslated region
